# *Drosophila* Nesprin-1 Isoforms Differentially Contribute to Muscle Function

**DOI:** 10.3390/cells10113061

**Published:** 2021-11-06

**Authors:** Alexandre Rey, Laurent Schaeffer, Bénédicte Durand, Véronique Morel

**Affiliations:** 1 LBMC, ENS de Lyon, CNRS UMR5239, 69007 Lyon, France; arey38920@gmail.com; 2 Institut NeuroMyoGène, CNRS UMR5310, INSERM U1217, Université Claude Bernard Lyon 1, 69008 Lyon, France; laurent.schaeffer@univ-lyon1.fr (L.S.); benedicte.durand@univ-lyon1.fr (B.D.)

**Keywords:** Nesprin-1, Msp300, *Drosophila*, myofibrils, desmin, isoforms, nuclei clustering, isoform

## Abstract

Nesprin-1 is a large scaffold protein connecting nuclei to the actin cytoskeleton via its KASH and Calponin Homology domains, respectively. Nesprin-1 disconnection from nuclei results in altered muscle function and myonuclei mispositioning. Furthermore, Nesprin-1 mutations are associated with muscular pathologies such as Emery Dreifuss muscular dystrophy and arthrogryposis. Nesprin-1 was thus proposed to mainly contribute to muscle function by controlling nuclei position. However, Nesprin-1′s localisation at sarcomere’s Z-discs, its involvement in organelles’ subcellular localization, as well as the description of numerous isoforms presenting different combinations of Calponin Homology (CH) and KASH domains, suggest that the contribution of Nesprin-1 to muscle functions is more complex. Here, we investigate the roles of Nesprin-1/Msp300 isoforms in muscle function and subcellular organisation using *Drosophila* larvae as a model. Subsets of Msp300 isoform were down-regulated by muscle-specific RNAi expression and muscle global function and morphology were assessed. We show that nuclei anchoring in mature muscle and global muscle function are disconnected functions associated with different Msp300 isoforms. Our work further uncovers a new and unsuspected role of Msp300 in myofibril registration and nuclei peripheral displacement supported by Msp300 CH containing isoforms, a function performed by Desmin in mammals.

## 1. Introduction

Skeletal muscle cells are large elongated syncytia characterised by a central contractile apparatus, presenting a semi-crystalline organisation of actin and myosin filaments, and peripheral nuclei evenly spaced along the fibre length with the exception of a few sub-synaptic nuclei grouped under the neuromuscular junction. Surface localisation and regular spacing of myonuclei is a hallmark of mature muscle fibres and is preserved despite the intense cytoplasmic flow generated by muscle contraction. This organisation appears key to normal muscle function since centrally located or clustered nuclei have been associated with several muscular pathologies including Emery Dreifuss muscular dystrophy and arthrogryposis [1].

Particular attention has been paid to Nesprin proteins as important players of myonuclei organisation. Nesprins (nuclear envelope spectrin repeat proteins) are scaffold and linker proteins with a conserved organisation of their protein domains among species. They are characterised by C-terminal KASH (Klarsicht/ANC-1/Syne Homology) domain mediating their anchorage in the nuclear envelope and a central rod of Spectrin Repeats (SR). Nesprin-1 and -2, the largest of the four mammalian Nesprins, are further characterised by two Calponin Homology (CH) domains found at their N-terminus and interacting with the actin cytoskeleton. For both proteins, several isoforms, resulting from the use of different start exons as well as alternative splicing, have been described [2,3,4]. In muscle, the main Nesprin isoforms are the giant Nesprin (gNesp), corresponding to the full-length protein, and Nesprin-α (Nesp-α), truncated from the N-terminal part of the protein and lacking the CH domains [5,6]. Their expression is modulated during muscle maturation: gNesp-1, the main isoform in immature muscle fibres, being gradually replaced by the Nesp-1α isoform as muscle fibres mature [7].

Nesprin-1 has been shown to contribute to several aspects of muscle fibres’ development and functions. While a lot of knowledge has been gathered on the contribution of Nesprin-1 isoforms to the organisation of the myonuclei in mammalian muscle, a lot remains to be discovered concerning the other muscular functions of the Nesprin-1 isoforms.

During myogenesis, myoblast fusion is accompanied by a complex ballet of nuclei which first concentrate at the centre of the forming fibre before adopting a regular spacing along the fibre’s length. This nuclei spreading is triggered by microtubules and microtubule motors [8] which are recruited to the nuclear envelope by Nesprin-1α and PCM-1 [9]. In agreement with such function, Nesprin-1 is found at the nuclear envelope of myonuclei, gNesp-1 being evenly localised around the nuclei while Nesp-1α2 concentrates at the surface of the nuclear envelope facing the neighbouring nucleus [10]. Finally, as myofibrils form, myonuclei move to the periphery of the fibre, in the narrow cytoplasmic space between the plasma membrane and the contractile apparatus. In mice, deletion of the KASH domain results in the disconnection of Nesprin-1 from the nuclear envelope [11,12,13] as well as muscle weakness and respiratory failure [13,14,15]. These observations, and the identification of numerous Nesprin-1 mutations linked to Emery Dreifuss muscular dystrophy, arthrogryposis and dilated cardiomyopathies [16] led to the proposal that Nesprin-1′s main muscular function is to anchor nuclei, disruption of nuclei anchorage and subsequent myonuclei mislocalisation being causal to these pathologies.

In addition to its contribution to myonuclei organisation, Nesprin-1 was found as a Golgi enriched protein [17,18]. Mutants of ANC-1 or Msp300, C. elegans and *Drosophila* Nesprin-1 respectively, were also associated with ER and mitochondria mispositioning [19,20] and Nesprin-1 and Msp300 have been shown to localise at Z-discs in sarcomeres [13,19,21]. Finally, Msp300 and CPG2 (a short mammalian Nesprin-1 isoform) have been involved in neuromuscular junction plasticity and homeostasis control [21,22,23].

The diversity of Nesprin-1′s roles in muscles as well as the large number of Nesprin-1 isoforms identified suggest that Nesprin-1′s muscular functions could be supported by different Nesprin-1 isoforms. Here, we investigate the relative contribution of Nesprin-1/Msp300 isoforms to muscle functions in *Drosophila* larva. We show that nuclei positioning and global muscle function are disconnected functions that can be attributed to different Msp300 isoforms. Our work further uncovers a new and unsuspected role of Msp300 in myofibril registration supported by Msp300 giant isoforms.

## 2. Materials and Methods

### 2.1. Genetics

*Drosophila* stocks were obtained or modified from stock from the following sources:

;Msp300-Venus/CyO,Dfd-YFP;; (w1118;PBac{768.FSVS-0}Msp300CPTI003472/SM6a;;, Kyoto Stock Center), ;;24B-Gal4; (Bloomington Stock Center), w1118;P{GD8201}v17346;; (UAS-ex2[RNAi]), w1118;;P{GD9539}v40143; (UAS-ex17[RNAi]), w1118;P{KK112156}v107183;; (UAS-ex23[RNAi]), w1118;;P{GD10406}v25906; (UAS-ex28[RNAi]), w1118;P{GD936}v51446;; (UAS-lacZ[RNAi]) from the Vienna Drosophila Resource Centre. UAS-[RNAi] lines were combined with UAS-Dicer2 transgene to improve RNAi efficiency, except for UAS-lacZ[RNAi]. Canton S were used as wild-type (WT) control for locomotion assays and RT-qPCR. For RNAi experiments, females carrying the Gal4 transgene were crossed to RNAi males. Flies were raised in a natural daylight cycle on classical cornmeal agar food. For locomotion assays, cages were set up with agar plates with some active yeast paste. Eggs were laid for 2 h and then aged at 25 °C before being processed.

### 2.2. Locomotion Assays

For the global locomotion assay, 48 h AEL (after egg laying) old larvae were collected and washed in water. A single larva was positioned at the centre of an agar plate placed above a 5 × 5 mm grid. Locomotion was tracked manually under the microscope (Discovery.V8, Zeiss, (Jena, Germany)) for 2 min and we scored the number of squares crossed by the larva. A picture of the larva was taken using a camera (AxioCam MRc5, Zeiss) coupled to the microscope and Axiovision software was used to measure larval size. The global locomotion score was calculated as follows: Nb(squares) × 5000/larval size (in µm). Locomotion tests were performed at 22–23 °C. In order to control for external stimuli, each assay included a group of control wild-type larvae used as a reference for the behavioural analysis. Independent locomotion assays were repeated as follows: ex2[RNAi]: 8 repeats with 7 or 8 larvae each; ex17[RNAi]: 9 repeats with 7 or 8 larvae each; ex23[RNAi]: 10 repeats with 7 or 8 larvae each; ex28[RNAi]: 9 repeats with 7 or 8 larvae each. All tests included a group of 7 or 8 control larvae.

### 2.3. Immunostainings

Wandering L3 larvae were dissected in lab-made HL3.1 Ca2+ free medium [21,22] and fixed 20 min in 4% paraformaldehyde (PFA) in Phosphate Buffer Saline (PBS) 1× as described previously [22]. Briefly, third instar wandering larvae were collected and washed in PBS ×. They were pinned on a silicon plate at their anterior and posterior extremities and opened dorsally. The cuticle and the attached muscles were stretched and pinned on the side and the opened and flatten larvae were fixed in 4% PFA for 20 min. Larvae were extensively washed in PBS1X- 0.1% Triton X-100 (PBTx), blocked for 1 h in PBTx-1% BSA (PBTx-BSA) and incubated overnight at 4 °C with the primary antibodies. After several washes in PBTx-BSA, secondary antibodies and phalloidin were added for 2 h at RT. After extensive washes, the larvae were mounted in Vectashield (VectorLab). Anti-Kettin (rat, MAC 155, 1/200) was obtained from Babraham Institute and Developmental Studies Hybridoma Bank provided for anti-LaminA (mouse, Lamin Dm0, ADL67.10, P. Fischer, 1/200). phalloidin-Alexa555 (1/400) was purchased at Interchim, secondary antibodies were: anti-mouse-Alexa488 from Molecular Probe and anti-rat-Alexa647 (1/1000) from Jackson ImmunoResearch Europe. In all the experiments, larvae of the different genotypes were processed identically and simultaneously.

### 2.4. Imaging

For nuclear clustering quantification, we acquired images of muscles 4, 6 and 7 from abdominal segments 3 and 4 with a Zeiss Plan neoFluar 20×/0.5 objective mounted on an AxioImager Z1 microscope (Zeiss) combined with a Coolsnap HQ camera (Photometric) and controlled by Metamorph software (Molecular Devices, San Hoze, CA, USA). Other immunofluorescent images were acquired with a Leica spectral confocal (DM 6000B microscope combined with an SP5 scanning device and an HCx PlanApo 60×/1.4 oil immersion objective from Leica) controlled by the Leica LAS-AF software. We used an immersion oil type LDF (index 1.515) and acquisitions were performed at 22 °C.

### 2.5. Image Analysis

For nuclear clustering, nuclei were scored as part of the same cluster when they had contacting nuclear envelopes. The percentage of clustering corresponds to the number of nuclei in a given cluster’s size to the total number of nuclei in that fibre.

For Z-disc defects, 90 to 200 Z-discs from 3 to 4 muscle fibre 4 from distinct larvae were analysed.

### 2.6. RT-qPCR

We collected 20–23 h AEL old staged embryos expressing an individual RNAi line under the control of the pan-muscular driver 24B-Gal4, then they were washed in water and ground in Trizol (Invitrogen, Waltham, MA, USA) for RNA extraction and purification. For each sample, 300 ng RNA, first treated by the TurboDNAse (Ambion, Waltham, MA, USA), were reverse transcribed using random hexamers (ThermoScientific, Waltham, MA, USA) and MMLV-H reverse transcriptase (Fermentas, Waltham, MA, USA). Real-time PCR (Rotor Gene) was performed using primer couples overlapping Msp300 exon–exon boundaries between the RNAi targeted exon and its immediate neighbour and SYBR green (Qiagen) for amplicon detection and quantification. Msp300 exon boundaries used were ex2–ex5, ex16–ex17, ex23–ex24 and ex28–ex30 (see Supplementary Figure S1 for localisation and Supplementary Table S1 for sequence). *tbp-1* served as the reference gene. For comparison purposes, WT and UAS-RNAi/+ and 24B-Gal4/+ samples were always processed simultaneously. Relative mRNA levels were quantified as described in [23].

### 2.7. Statistics and Graphical Representations

The Wilcoxon statistical test was performed with R. Box plots were done using 10th and 90th percentiles.

## 3. Results

**Figure 1:**
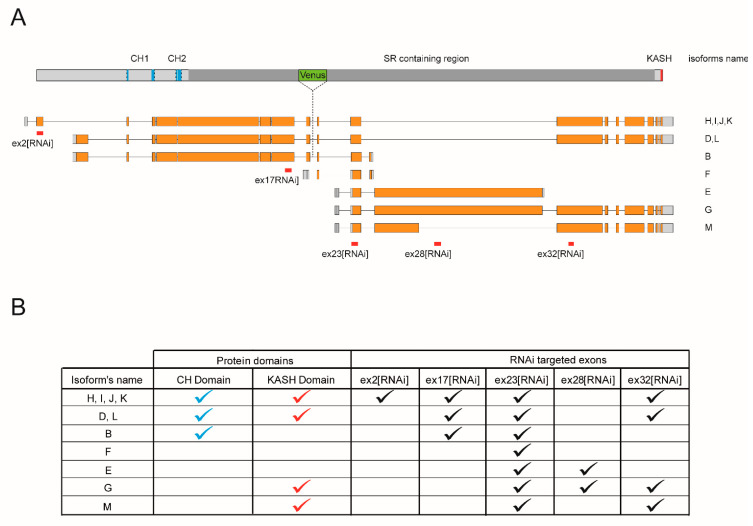
Msp300 isoforms. (**A**) Schematic representation of Msp300 isoforms. Isoform diversity results from the use of different start and stop exons (respectively exon 1, 3, 18 and 21 for the start and exon 26, 29, 40 and 41 for the stop) but also from alternative splicing events. For simplicity, isoforms H, I, J and K, all starting at exon1 and containing the C-terminal KASH domain have been represented as a single transcript despite the use of few alternative exons within the coding sequence (top transcript). Similarly, isoforms D and L differ by one alternative splice event and have been represented by a single transcript (second top transcript). Isoform M is represented here although RNAseq tissue-specific analysis suggests that it is not expressed in larval muscle [27]. Exon structure is represented at scale (coding region in orange, non-coding in grey). RNAi amplicons are shown in red under the targeted exon. ex32[RNAi] was used in a previous work and positioned here for discussions sake. Protein features are positioned above the isoforms schemes: Calponin Homology domains (CH) are shown in blue, KASH domain in red, the region containing the Spectrin Repeats (SR) in dark grey and the protein-trap transposon used in this study in green (based on Flybase, http://flybase.org/reports/FBgn0261836, accessed on 6 May 2021). (**B**) Table summarising the structure of isoforms and RNAi targets.

Msp300 was first thought to be a dystrophin-like protein [24] before bioinformatic analysis [25] and biological evidence [26] identified it as the only *Drosophila* ortholog of Nesprins more closely related to Nesprin-1. Eleven Msp300 isoforms have been postulated as the result of the combined use of four transcription start sites, at exon1, 3, 18 and 21, 4 poly-adenylation signals and alternative splice sites. These postulated isoforms can be grouped into 4 categories based on the presence or absence of the N-terminal Calponin Homology or the C-terminal KASH domains (Figure 1A,B).

This repertoire of Msp300 isoforms presents similarities with mammalian Nesprin-1 and 2 described isoforms [4,6]. As for mammalian Nesprin-1 and 2, one can identify long Msp300 isoforms containing both CH and KASH domains (D, L, H, I, J and K; CH+KASH+), but also truncated isoforms containing either domains or none. Isoform B is the only predicted isoform containing the CH but not the KASH domains (CH+KASH-), while six Nesprin-1 mammalian isoforms have been proposed to present such CH+KASH- organisation [4]. On the opposite, Msp300 isoforms M and G lack CH but include KASH domains (CH-KASH+), similar to the Nesprin-1α structure. In addition, two isoforms have been proposed to contain neither CH nor the KASH domain: the short isoform F, containing a single Spectrin Repeat, and isoform E. Isoforms E, M and G further partially share exon28, which is absent from all other isoforms. Msp300 isoforms thus appear to be cognate with Nesprin-1 and 2 isoforms.

Previous studies [19,26] have shown the global contribution of Msp300 to different subcellular aspects of muscle function, including larval locomotion, myonuclei localisation but also localisation of Msp300 at the Z-disc. Here, we investigated the specific contribution of the different Msp300 isoforms to muscle function in *Drosophila* larvae, focusing on CH containing versus CH lacking isoforms.

Because of the large size of Msp300 isoform transcripts (between 21 and 43 kb for all but isoform F) and the complexity of the locus (41 exons shared by several isoforms), it is not possible to directly study individual isoforms. We therefore chose to use a few RNAi lines specifically targeting relevant isoform groups and infer from the differences in phenotypes the potential functions of Msp300 isoforms. We took advantage of UAS-RNAi lines generated by the VDRC consortium that rely on the use of the UAS/Gal4 system for their expression [28] to target different Msp300 exons (they are hereafter named after the targeted exon, Figure 1A). Since all exons are shared by several isoforms, these tools were used to target isoform subsets. ex32[RNAi] targets all KASH containing isoforms and was previously used to establish Msp300′s contribution to the control of post-synaptic glutamate receptor density [26]. In this study, we extended our analysis to discriminate between CH+ and CH- Msp300 isoforms: ex23[RNAi], which targeted exons, is shared by all Msp300 isoforms and was used to down-regulate all isoforms, while ex17[RNAi] and ex2[RNAi] target all or a subset of CH containing isoforms. Finally, ex28[RNAi] was used to down-regulate CH-less isoforms E and G (summarised in Figure 1B).

**Figure 2:**
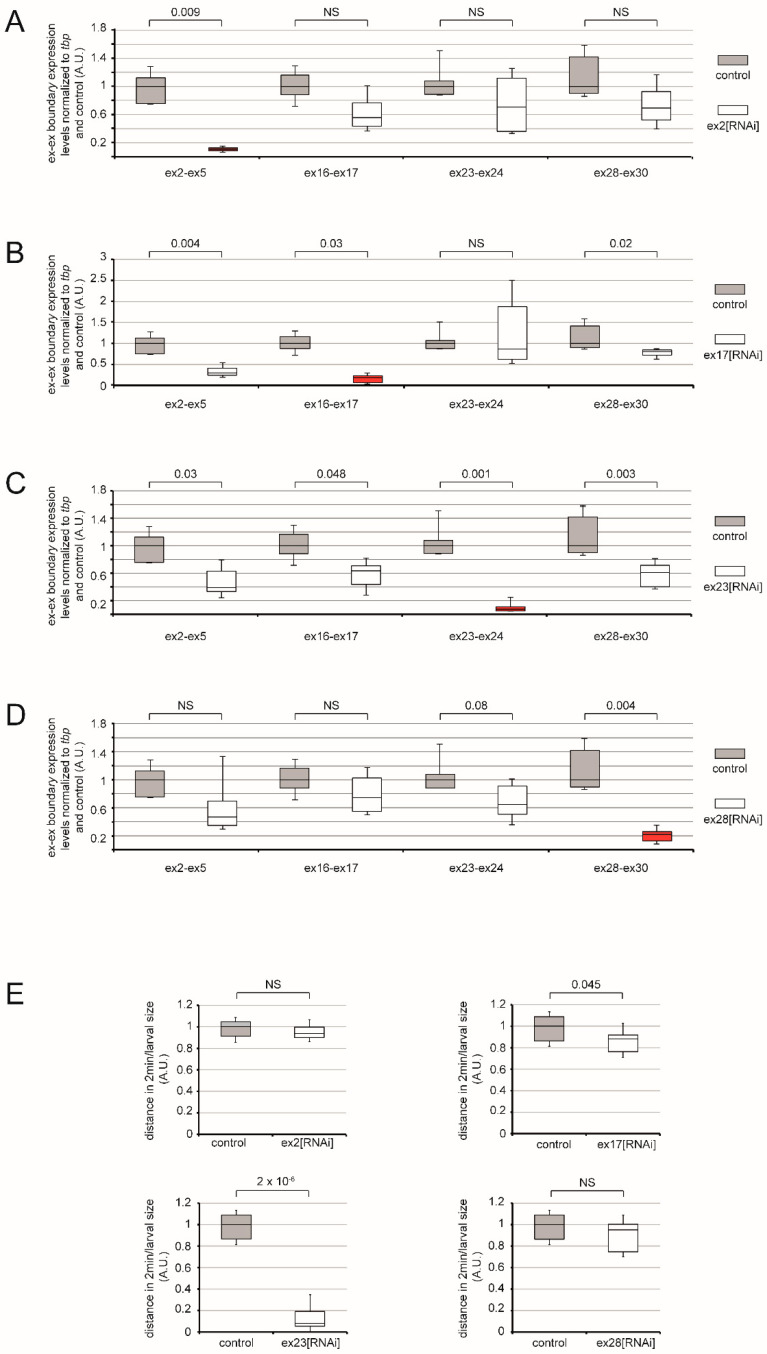
Msp300 isoforms differentially contribute to larval locomotion. (**A**–**D**) Box plot representation of muscular Msp300[RNAi] efficiency and impact on non-targeted isoform groups by RT-qPCR analysis. The expression level of each isoform group was measured using primers in the exon targeted by the specific Msp300[RNAi] and one neighbouring exon (primer localisation shown in Figure S1). For each primer couple and RNAi condition, mRNA levels were normalised to control larvae to allow comparison between the different conditions. For a given RNAi, expression levels of all isoform groups were tested. Box plots of the RT-qPCR assessing the RNAi effect on its targeted exon are shown in red, box plots corresponding to the non-targeted isoform subsets are in black. (**A**) ex2[RNAi], (**B**) ex17[RNAi], (**C**) ex23[RNAi], (**D**) ex28[RNAi]. (**E**) Box plot representation of the global displacement of 48 h AEL old larvae following 2 min of wandering relative to larval size. For comparison purposes between the different RNAi lines, muscle expressing Msp300[RNAi] larvae locomotion is normalised to respective control larvae locomotion. For each condition, one representative experiment is shown (WT, *n* = 7, *ex2[RNAi]*, *n* = 8, 8 repeats; WT, *n* = 15, *ex17[RNAi]*, *n* = 14, 7 repeats; WT, *n* = 15, *ex23[RNAi]*, *n* = 16, 7 repeats; WT, *n* = 15, *ex28[RNAi]*, *n* = 14, 7 repeats).

We first assessed by RT-qPCR the efficiency of RNAi knock-down by these 4 lines following pan-muscle expression in late *Drosophila* embryo, achieved with the use of a 24B-Gal4 driver. For all tested lines, mRNA levels of Msp300 isoforms were significantly and specifically knocked-down when compared to control (Figure 2A–D). Knock-down efficiency depended on the RNAi used: targeted mRNA levels were decreased to 10% of the control for ex2[RNAi], 18% for ex17[RNAi], 7% for ex23[RNAi] and 22% for ex28[RNAi].

In order to evaluate a possible indirect effect of RNAi on the other isoform’s expression levels and potential compensation effects induced by a given RNAi, we quantified the expression level changes of all isoform subsets systematically in all RNAi conditions tested. As shown in Figure 2C, targeting all isoforms with ex23[RNAi] results in a significant decrease in the expression level of all tested transcripts. On the opposite, down-regulation of ex2 only decreased ex2 containing transcripts to a significant level (Figure 2A), while down-regulation of ex17 containing isoforms, which include ex2 and ex3 containing isoforms, led to a decrease in the number of transcripts containing the ex16–ex17 boundary but also the ex2–ex5 boundary (Figure 2B). It also induced a small but significant decrease of ex28–ex30 containing transcripts. Finally, down-regulation of ex28 did not impact CH containing isoforms but only resulted in the decrease in the common ex23–ex24 boundary and the targeted ex28–ex30 boundary (Figure 2D).

RNAi conditions used here thus specifically target the desired isoform group without inducing compensation effect by the other isoforms and the muscular expression of these RNAi, and thus result in strong hypomorphic conditions for the different groups of Msp300 isoforms.

### 3.1. Msp300 Isoforms Differentially Contribute to Larval Locomotion

**Figure 3:**
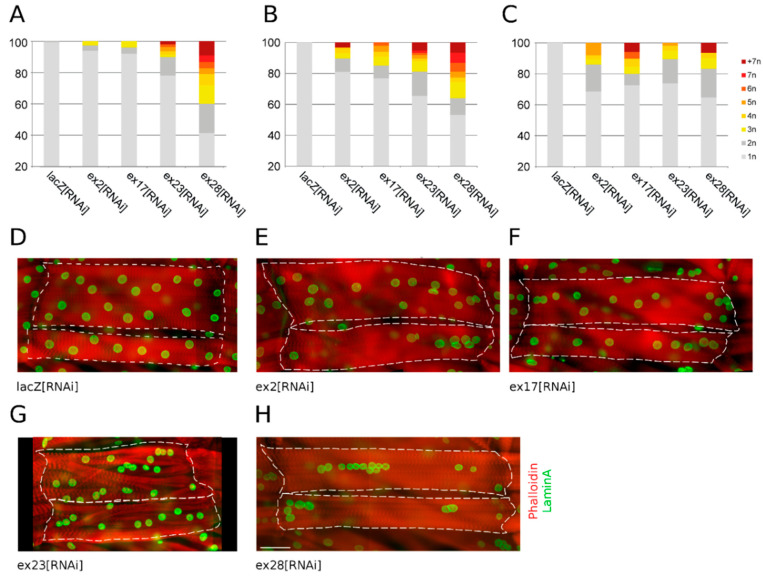
Msp300 isoform depletion differentially alters nuclei positioning in the muscle. (**A**–**C**) Nuclei clustering quantification in fibres 4 (**A**), 6 (**B**) and 7 (**C**), comparing the effects of muscular expression of either lacZ[RNAi] or Msp300[RNAi] and showing the percentage of total nuclei found isolated or in groups of two to more than seven nuclei. Percentage of nuclei involved in clusters and size of the clusters vary with the RNAi and the muscle type used. Number of scored larvae and total number of counted nuclei are: lacZ[RNAi]: 9 larvae, 402/483/281nuclei (F4/F6/F7); ex2[RNAi]: 7 larvae, 244/326/188 nuclei; ex17[RNAi]: 6 larvae, 186/263/143 nuclei; ex23[RNAi]: 11 larvae, 357/416/259 nuclei; ex28[RNAi]: 9 larvae, 352/402/246 nuclei. (**D**–**H**) Immunostaining of muscles 6 and 7 expressing lacZ[RNAi] (**D**), ex2[RNAi] (**E**), ex17[RNAi] (**F**), ex23[RNAi] (**G**), ex28[RNAi] (**H**) showing representative nuclei clustering phenotypes. Nuclei labelled with LaminA antibody (green) and contractile apparatus with phalloidin (red). Fibres are outlined with white dotted lines for ease of identification. In each panel, Fibre 6 on top and 7 below, anterior left. Scale bar on ex28[RNAi] panel: 100 μm.

*Drosophila* larva locomotion is commonly used as a means to investigate global muscle function [29]. We previously performed locomotion assays on stage 2 larvae (L2), measuring the distance crawled by larvae in 2 min normalised to their size (global locomotion assay) and showed that deletion of Msp300 KASH domain or knock-down of KASH containing isoforms following ex32[RNAi] expression results in severe larval locomotion defects (respectively less than 30% and 22% of the control, Figure 2A and Figure 3A, in [26]). Here we investigated the contribution of CH- versus CH+ Msp300 isoforms to muscle function by monitoring larval locomotion upon muscular expression of the different RNAi. We first down-regulated all Msp300 isoforms by expressing ex23[RNAi] in larval muscles and observed a strong locomotion defect (Figure 2E, the best score of ex23[RNAi] expressing larvae corresponding to 18% of the score of control larvae, *p* = 2 × 10^−6^), in agreement with our published results and the wide spectrum of Msp300 targeted isoforms by this RNAi. We next targeted CH+ isoforms with ex17[RNAi] muscular expression and we observed a mild but significant larval locomotion decrease (82% of the control, *p* = 0.045), while ex2[RNAi] expression did not result in larval locomotion defects. The difference between ex17 and ex2[RNAi] could reveal either the central role of CH+ isoforms B, D and L in locomotion or the capacity of these isoforms to compensate for isoforms H, I, J and K down-regulation. Finally, we did not observe any locomotor impairment following down-regulation of CH-isoforms Msp300-G and -E by ex28[RNAi] muscular expression.

While knocking-down all KASH+ or all CH+ Msp300 isoforms led to locomotion defects, knock-down of CH- Msp300 isoforms is not associated with impairment of locomotion. Hence, we reveal here a differential requirement of CH+KASH+ versus CH- Msp300 isoforms for larval locomotion.

### 3.2. Nuclei Clustering and Larval Locomotion Are Disconnected Phenotypes

Mammalian studies have highlighted a strong correlation between muscular defects and myonuclei clustering after Nesprin-1 and 2 mutations. In *Drosophila*, deletion of Msp300 KASH domain, as well as deletion of its C-terminal half, have been associated with both muscular dysfunction and nuclei clustering [19,26]. We therefore evaluated the contribution of the different subsets of Msp300 isoforms to myonuclei localisation.

For each RNAi condition, we counted the percentage of nuclei distributed in clusters (i.e., nuclei touching each other). These were scored in three longitudinal muscles: muscle 4, 6 and 7 (Figure 3A–C respectively, [30]). The total per cent of clustered nuclei as well as clustering strength, as measured by the number of nuclei per cluster, varied with the RNAi and the muscle fibre considered. When targeting all Msp300 isoforms with ex23[RNAi] we observed nuclei clustering in all muscles scored (Figure 3A–C,G), with 22 to 35% of clustered nuclei, including 2 to 10% of nuclei involved in clusters of 5 or more nuclei. We next investigated the contribution of the different subsets of isoforms. When knocking-down CH containing isoforms we observed a moderate clustering (Figure 3A–C,E,F), while down-regulation of CH- isoforms led to a very strong and penetrant clustering phenotype (Figure 3A–C,H), with 35 to 59% of nuclei clustered out of which more than 20% were found in large clusters in fibres 4 and 6.

Thus, knock-down of all Msp300 isoforms results in nuclei clustering. Unexpectedly, although ex23[RNAi] is postulated to target all isoforms, the associated clustering phenotype is intermediate between ex2[RNAi], ex17[RNAi] and ex28[RNAi] associated phenotypes. Furthermore, although ex28[RNAi] larvae do not present locomotion defects, they are characterised by a strong clustering phenotype. Hence our observations demonstrate that nuclei clustering can be disconnected from muscle locomotor function.

### 3.3. Knock-Down of Msp300 Isoforms Alters Shape and Surface Localisation of Nuclei

**Figure 4:**
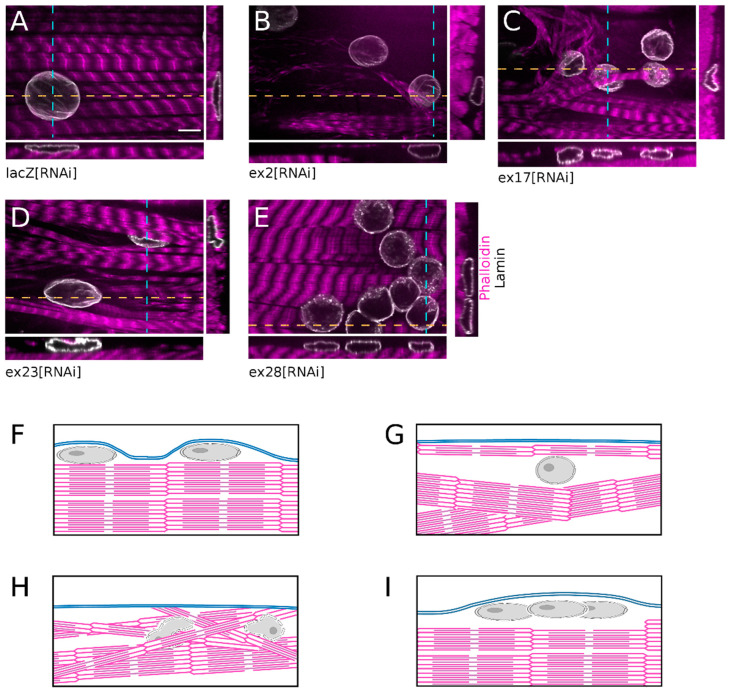
Nuclei fail to reach the contractile apparatus surface upon Msp300 isoform depletion. (**A**–**E**) Immunostaining of muscle 4 expressing lacZ[RNAi] (**A**), ex2[RNAi] (**B**), ex17[RNAi] (**C**), ex23[RNAi] (**D**) and ex28[RNAi] (**E**) showing nuclei position with respect to the contractile apparatus (respectively labelled with anti-LaminA in grey and phalloidin in magenta). For each panel, orthogonal views of the muscle are shown on the side and below. XZ views are marked by a dashed orange line and shown below the panel while YZ views are marked by a dashed cyan line and shown on the right of the XY view. While nuclei are positioned at the periphery of the muscle (above the sarcomeres) in lacZ and ex28[RNAi] conditions, they are found intermingled with myofibrils in the other conditions. Orthogonal views show myofibrils above nuclei as well as the distorted shape of nuclei. For each orthogonal view, the muscle surface is either on the top (XZ view at bottom of each panel) or on the right (YZ view on left of each panel). Scale bar on lacZ[RNAi] panel: 10 μm. (**F**–**I**) Schematic representations of muscle orthogonal views showing the nuclei position with respect to myofibrils and the plasma membrane in lacZ[RNAi] (**F**), ex2[RNAi] (**G**), ex17 or ex23[RNAi] (**H**) and ex28[RNAi] (**I**). Nuclei are shown in grey, plasma membrane in blue and myofibrils in magenta.

While monitoring myonuclei localisation we observed that all clustered nuclei following ex28[RNAi] were localised at the surface of the fibre, within thin cytoplasmic domains, and had a flat regular round shape (Figure 4E,I), like in control lacZ[RNAi] fibres (Figure 4A,F). In comparison, ex2[RNAi] nuclei were not as flat and were often surrounded by large cytoplasmic domains devoid of myofibrils. In addition, the contractile apparatus presented occasional disorganised myofibrils (Figure 4B,G). The organisation of muscles after ex17[RNAi] was even more perturbed. Nuclei were often trapped below disorganised myofibrils, close to the muscle surface, and presented an irregular shape with frequent protrusions (Figure 4C,H). The ex23[RNAi]-associated phenotype was the most severe. In addition to nuclei surrounded by myofibrils-free cytoplasmic domains, we observed nuclei trapped in the middle of the contractile apparatus and which presented an irregular or folded nuclear shape (Figure 4D,H).

Thus, down-regulation of Msp300 isoforms alters myonuclei organisation within the muscle. CH-KASH+ isoforms are required for the correct spacing of nuclei once they have reached the cell surface but do not impact the nuclei outward migration. On the opposite, knock-down of CH containing Msp300 isoforms interferes with nuclear displacement to the muscle surface, resulting in centrally located nuclei.

### 3.4. Msp300 Isoforms RNAi Impair Myofibril Registration

Myogenesis involves two sequences of nuclear movements. First, during myoblast fusion, the nuclei coalesce at the centre of the syncytium before spreading along the length of the fibre to maximise internuclei distances [8,31,32,33,34]. Then, myonuclei move to the periphery of the fibre and localise at the surface of the contractile apparatus, in a narrow space between the sarcomeres and the plasma membrane. This displacement has been shown to be the consequence of expelling forces applied on nuclei by myofibrils as they assemble to form a coherent contractile apparatus, a phenomenon called myofibril registration. Interestingly, myofibril registration involves Z-discs zipping mediated by Desmin, mutations of which result in nuclei trapped within the contractile apparatus and presenting an irregular shape [35]. This is reminiscent of our observation of trapped nuclei following the knock-down of CH+ Msp300 isoforms. We thus investigated the consequences of Msp300 isoforms knock-down on Z-disc, sarcomere and Msp300 subcellular organisation.

**Figure 5:**
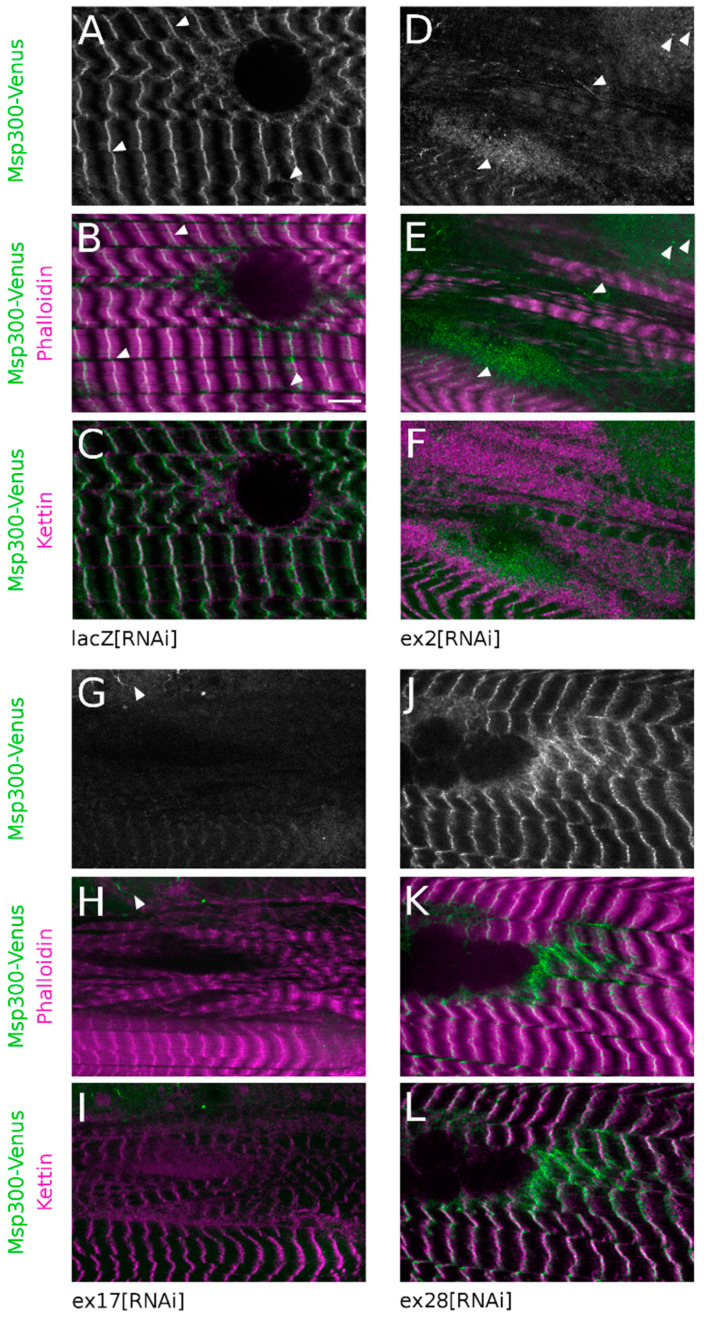
Depletion in CH containing Msp300 isoforms results in disorganisation of the myofibrils. Muscle 4 expressing Msp300-Venus protein-trap together with lacZ[RNAi] (**A**–**C**), ex2[RNAi] (**D**–**F**), ex17[RNAi] (**G**–**I**), or ex28[RNAi] (**J**–**L**) and showing the contractile apparatus organisation. (**A**,**D**,**G**,**J**): Msp300-Venus localisation. (**B,E,H,K**): phalloidin labelling in magenta and Msp300-Venus fluorescence in green. (**C,F,I,L**): Kettin immunostaining in magenta and Msp300-Venus fluorescence in green. In control lacZ[RNAi] (**A**–**C**), sarcomeres present a regular organisation with alternate (**A**)–band, lacking phalloidin staining, and Z-discs, showing bright phalloidin staining (**B**). Myofibrils are registered as seen with the large domains of sarcomeres’ alignment. Msp300-Venus ((**A**) in grey, (**B**,**C**) in green) presents a typical “beads-on-string” pattern (arrow heads) and localises at Z-discs as shown with the co-localisation with Kettin antibody ((**C**), in magenta). This organisation is conserved following ex28[RNAi] (**J**–**L**) muscular expression. Msp300-Venus “beads-on-string” labelling at Z-discs is largely lost following ex2[RNAi] although occasional short Msp300-Venus strings can still be observed ((**D**,**E**), arrow heads). Msp300-Venus disorganisation is even stronger following ex17[RNAi] (**G**,**H**) with a complete loss of the bead pattern at Z-discs and only weak and rare Msp300-Venus staining in the cytoplasm (arrow head). Myofibril registration is also strongly perturbed following ex2, ex17[RNAi] but sarcomere structure, as seen with the regular Kettin/Z-disc pattern, is mostly preserved (respectively (**F**) and (**I**)). Scale bar on lacZ[RNAi] panel: 10 μm.

Kettin/alpha-actinin, a constitutive component of Z-discs, was used as a marker of Z-disc integrity and a Venus protein-trap inserted between exon19 and 20 (Figure 1A, Msp300-Venus) was used to follow the subcellular localisation of CH+ Msp300 isoforms (but not CH- isoforms, starting at exon21 and thus not tagged by Venus). As previously described for Msp300 [26], in control fibres Msp300-Venus is organised in long strings of beads in the cytoplasm surrounding nuclei at the edge of muscle fibres. These strings also localise at Z-discs and between Z-discs where they appear as connectors between myofibrils (Figure 5A–C, arrow heads). This organisation is not modified following ex28[RNAi] expression, confirming that Msp300-Venus does not tag exon28 containing isoforms (Figure 5J–L). We next investigated the consequences of down-regulating CH+ isoforms on Msp300-Venus localisation. Targeting all Msp300 isoforms with ex23[RNAi] resulted in a complete loss of Msp300-Venus labelling both in the cytoplasm and at sarcomeres (Figure S2I). When down-regulating all CH+ isoforms with ex17[RNAi], Msp300-Venus “beads-on-string” localisation at Z-discs was absent but rare Msp300-Venus punctae, organised in short strings, could still be observed in cytoplasmic pockets (Figure 5G–I, arrow heads). In agreement with the measured RNAi efficiency (Figure 2A), following ex2[RNAi] Msp300-Venus was occasionally seen as individual punctae, rarely organised in small strings, in cytoplasmic pockets and as weak and sparse punctae at Z-discs (Figure 5D–F, arrow heads). These results show that CH+ isoforms are targeted at Z-discs in muscle fibres.

We then studied the consequences of down-regulating Msp300 isoforms on the organisation of Z-discs and sarcomeres, monitored respectively with Kettin and phalloidin.

**Figure 6:**
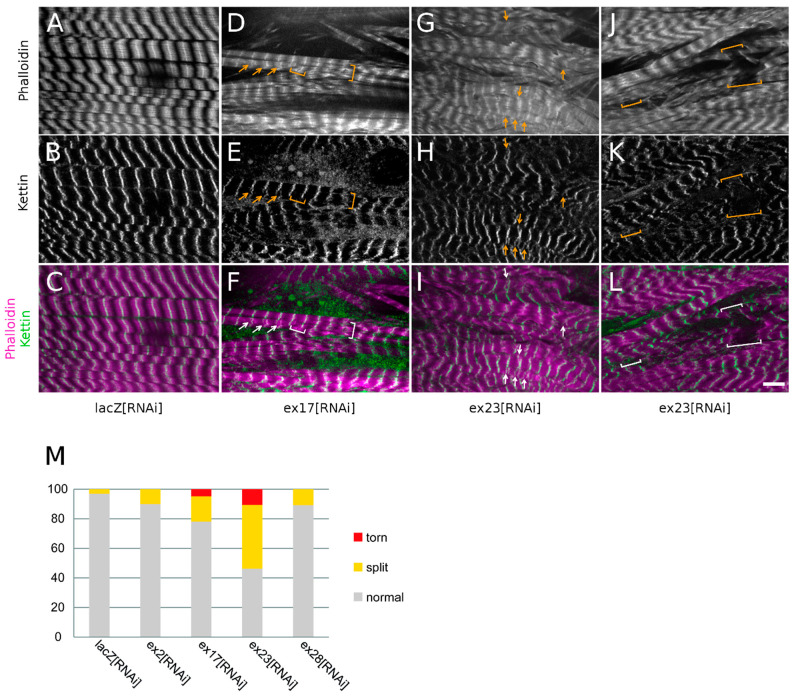
Depletion of CH containing Msp300 isoforms with ex17[RNAi] or ex23[RNAi] results in Z-discs weakening. (**A**–**L**) Muscle 4 from larvae expressing Msp300-Venus protein-trap together with lacZ (**A**–**C**), ex17[RNAi] (**D**–**F**) or ex23[RNAi] (**G**–**L**) and showing the contractile apparatus organisation. phalloidin labelling in grey (**A**,**D**,**G**,**J**) or magenta (**C**,**F**,**I**,**L**) and Kettin immunostaining in grey (**B**,**E**,**H**,**K**) or green (**C**,**F**,**I**,**L**). In addition to important disorganisation of the myofibrils, ex23[RNAi], and to a lesser extent ex17[RNAi], lead to splittings of the Z-discs (arrows in (**D**–**I**)) as well as ruptures of myofibrils (brackets in (**D**–**F**) and (**J**–**L**)). These ruptures occur at Z-discs, suggesting that CH+ Msp300 isoform depletion weakens Z-discs’ cohesion. (**M**) Quantification of Z-disc defects following Msp300[RNAi]. Scale bar: 10 μm.

CH- isoforms down-regulation with ex28[RNAi] did not alter the organisation of sarcomeres (Figure 5K) and Z-discs (Figure 5L, compare with control lacZ[RNAi], Figure 5B,C). These observations are in agreement with the correct peripheral localisation of myonuclei in ex28[RNAi] muscles. Similarly, genomic deletion of the Msp300 KASH domain did not induce myofibril disorganisation nor defects in the peripheral displacement of nuclei [26]. In contrast, down-regulation of CH+ Msp300 isoforms following ex2 or ex17[RNAi] resulted in partial alteration of the registration of myofibrils, with gaps between large contractile apparatus blocs and some disorganised myofibrils not registered with the rest of the contractile apparatus at the surface of the muscle (Figure 5D–I). These myofibrils nevertheless retained a regular organisation of sarcomeres, as seen with the alternate A-band (lacking phalloidin staining, Figure 5E,H) and the regular Kettin localisation in stripes (Figure 5F,I). Furthermore, ex23[RNAi] expression in muscle induced an exacerbated disorganisation of the contractile apparatus characterised by entangled and poorly registered myofibrils (Figure 6G–L).

When investigating sarcomeric organisation, we observed in ex23[RNAi] conditions frequent Z-disc splittings as shown by the widening of the bright Z-disc phalloidin labelling or the appearance of doubled or split Z-disc phalloidin lines (Figure 6G,I, arrows, 43% of Z-discs), and confirmed by the doubling of Kettin labelling (Figure 6H,I, arrows). ex23[RNAi] also resulted in torn myofibrils (brackets in Figure 6J–L) and we noticed that these breaks always occurred at the level of the Z-discs (Figure 6M, 11% of Z-discs). Z-disc splittings or breaks were also observed following ex17[RNAi] although to a lesser extent (Figure 6D–F,M, respectively 20% and 6% of Z-discs). ex2[RNAi] and ex28[RNAi] on the other hand only induced rare Z-disc splittings (10% of Z-discs) without Z-disc breaks (Figure 6M).

These observations suggest that Msp300 knock-down destabilises or weakens Z-disc organisation, fragilising myofibrils’ cohesion. The occurrence of Z-disc disorganisation with RNAi targeting all Msp300 isoforms or all CH+ isoforms but not with ex28[RNAi] further suggests that CH+ Msp300 isoforms strongly contribute to Z-disc cohesion.

In conclusion, CH containing Msp300 isoforms localise at Z-discs. We further show that knocking-down all CH containing but not CH-KASH+ Msp300 isoforms results in the alteration of the myofibril registration as well as the destabilisation of Z-discs.

## 4. Discussion

Here we investigated the differential contribution of Msp300 isoforms to larval muscle function and subcellular organisation. We show that Msp300 contributes to several aspects of muscle function: nuclei anchoring in the mature muscle fibre, muscle global function, nuclei peripheral migration, myofibril registration and Z-disc cohesion. We further attributed these different functions to different subsets of Msp300 isoforms.

### 4.1. Msp300 CH Containing Isoforms Are Important Players of Z-Discs’ Function in Muscle

Skeletal muscle fibres are large syncytia characterised by regularly spaced nuclei at their periphery. This cellular organisation is the result of complex nuclear movements occurring during the entire myogenesis process. During the initial steps of myotube formation, myonuclei coalesce at the centre of the cell before spreading along the entire myofiber’s length. As the contractile apparatus gets organised during muscle fibre maturation, the nuclei migrate at the periphery of the fibre where their regular spacing is maintained despite muscle contraction. This reorganisation is driven by both the contraction of the myofibrils and their cross-linking that allow sarcomeres to be registered and also squeeze the nuclei towards the surface [35].

Desmin is a key regulator in that process. Desmin is a muscle-specific intermediate filament localising at Z-discs. Mutations of *desmin* are associated with myofibrilar myopathies, a heterologous group of myopathies primarily affecting Z-disc organisation [36]. In mice, loss of *desmin* results in alteration of the lateral alignment of sarcomeres and in severely disrupted myofibrils with often split Z-discs [37]. It is also associated with isolated myofibers with distorted myonuclei encaged in myofibrils [35].

In *Drosophila*, the *desmin* gene is absent and a functional *drosophila* Desmin ortholog remains to be identified. However, we show here that Msp300 shares several features with Desmin. Msp300 localises at Z-discs and this localisation is lost upon down-regulation of CH containing isoforms. We furthermore observed deficient registration of myofibrils associated with distorted nuclei trapped between entangled myofibrils when we knocked-down CH containing Msp300 isoforms. Based on these observations, and since *Drosophila* lacks a functional *Desmin* ortholog, we propose that CH containing Msp300 isoforms either carry some of the Desmin functions during centrifugal nuclei movement or cooperate with a to-be-identified *drosophila Desmin* functional ortholog in that process.

Our experiments further reveal a weakness of myofibrils at the level of Z-discs which cohesion seems to be altered following the knock-down of all Msp300 isoforms. The observed phenotypes range from widened Z-discs to disrupted sarcomere links at Z-discs. These are reminiscent of phenotypes associated with mutations of Z-disc components ZASP or *Drosophila* filamin Cheerio which result in Z-discs widening and defective myofibrils alignment in adult flight muscle and were proposed to be required for Z-disc stability [38,39]. We thus propose that Msp300 could contribute to the stability of mature sarcomere and to their ability to sustain mechanical constraints associated with contraction–relaxation waves.

When interfering with CH-less isoforms, or in larvae carrying a genomic deletion of the Msp300 KASH domain [26], we did not observe deficient localisation of Msp300 at Z-discs, Z-disc cohesion, myofibrils registration or nuclei centrifugal relocation. In contrast, these phenotypes were always observed when knocking-down all CH containing Msp300 isoforms. We currently do not have the means to specifically deplete the CH+KASH-isoform among the CH+ isoforms. However, since depleting all KASH+ isoforms does not alter the registration of myofibrils, our results strongly support a role of CH+KASH- Msp300 isoform in Z-disc stability and registration, and subsequent nuclei displacement.

### 4.2. Msp300 CH-Less Isoforms Mediate Nuclear Anchoring but Are Dispensable for Larval Locomotion

When CH-less KASH containing Msp300 isoforms are down-regulated with ex28[RNAi], nuclei reach the muscle surface and adopt a flattened shape. They however fail to remain regularly spaced and cluster, often close to muscle fibres extremities. Similar phenotypes were observed with *Drosophila* genomic deletion removing either KASH domain coding sequences or all Msp300 sequence 3prime of exon 18, as well as with the down-regulation of KASH containing isoforms with ex32[RNAi] [19,26]. These observations thus suggest that both the KASH domain and exon28 are required for nuclei anchorage and lead us to speculate that nuclear anchoring in larval muscle is mediated by the Msp300-G isoform. These results are in agreement with previous observations in mice showing that long CH+KASH+ Nesprin-1 isoforms are dispensable for nuclear anchoring while specific deletion of the Nesprin-1α2 isoform lacking the CH domains results in nuclei clustering [40].

Stroud et al. (2017) [40] also observed perinatal lethality associated with Nesp1α2 deletion and concluded that this isoform is required for muscle function. Myonuclear clustering is also found in ageing muscles of Emery Dreifuss muscular dystrophy patients [41] and, in mice, genomic deletion of *Syne-1* (Nesprin-1 coding gene) results in nuclear clustering associated with decreased exercise ability [15]. Similarly, in *Drosophila*, the genomic deletion removing all Msp300 sequence 3prime of exon 18 is associated with both nuclei clustering and impaired larval locomotion [19,26]. These observations led to the proposal that nuclei clustering and altered muscle function are linked phenotypes.

We observed however that the knock-down of CH-less Msp300 isoforms leads to nuclei clustering with only a very mild alteration of muscle function. Conversely, the knock-down of CH containing isoforms (ex17[RNAi]) results in impaired larval locomotion but the nuclear localisation defects observed when depleting CH containing isoforms more likely reflect defective nuclei displacement towards the fibre’s periphery rather than defective nuclear anchoring. Based on our observations we thus propose that nuclei clustering and altered locomotion are disconnected phenotypes mediated by two pools of Msp300 isoforms: CH-KASH+ and CH+KASH- isoforms respectively. Because the genomic deletion removing the all Msp300 sequence 3prime of exon 18 or ex23[RNAi] impacts both pools of isoforms, they result in the combined nuclei clustering and larval locomotion phenotypes.

## 5. Conclusions

In conclusion, we show here that Msp300 isoforms differentially contribute to muscle homeostasis. We propose that CH-KASH+ Msp300 isoform G contributes to nuclear anchoring while CH containing isoforms, independently of the KASH domain, are involved in myofibril registration and nuclei displacement towards the muscle periphery, a so-far undescribed function of Msp300.

Future elucidation of Nesprin-1 isoforms specific functions, not only in muscle but also in other cell types including neurons, will allow understanding the mechanisms underlying the numerous Nesprin-1-associated pathologies including Emery Dreifuss muscular dystrophy and autosomal recessive cerebelar ataxia.

## Data Availability

modENCODE high-throughput RNA-seq data [27] available at http://www.modencode.org/ (accessed on 12 November 2021) or http://flybase.org/rnaseq/ (accessed on 12 November 2021).

## Supplementary Materials

The following are available online at https://www.mdpi.com/article/10.3390/cells10113061/s1, Figure S1: RT-qPCR primers position with respect to RNAi target exons; Figure S2: Msp300-Venus, phalloidin and Kettin localisation after isoforms depletion by RNAi; Table S1: RT-qPCR primers sequences.
