# Supplementary material for: Drosophila Nesprin-1 Isoforms Differentially Contribute to Muscle Function
Source: Cells. 2021 Nov 6;10(11):3061. doi: 10.3390/cells10113061 (PMC8616381; doi:10.3390/cells10113061)
Supplement: Supplementary file 1 [file cells-10-03061-s001.zip › cells-1431556-supplementary.pdf]

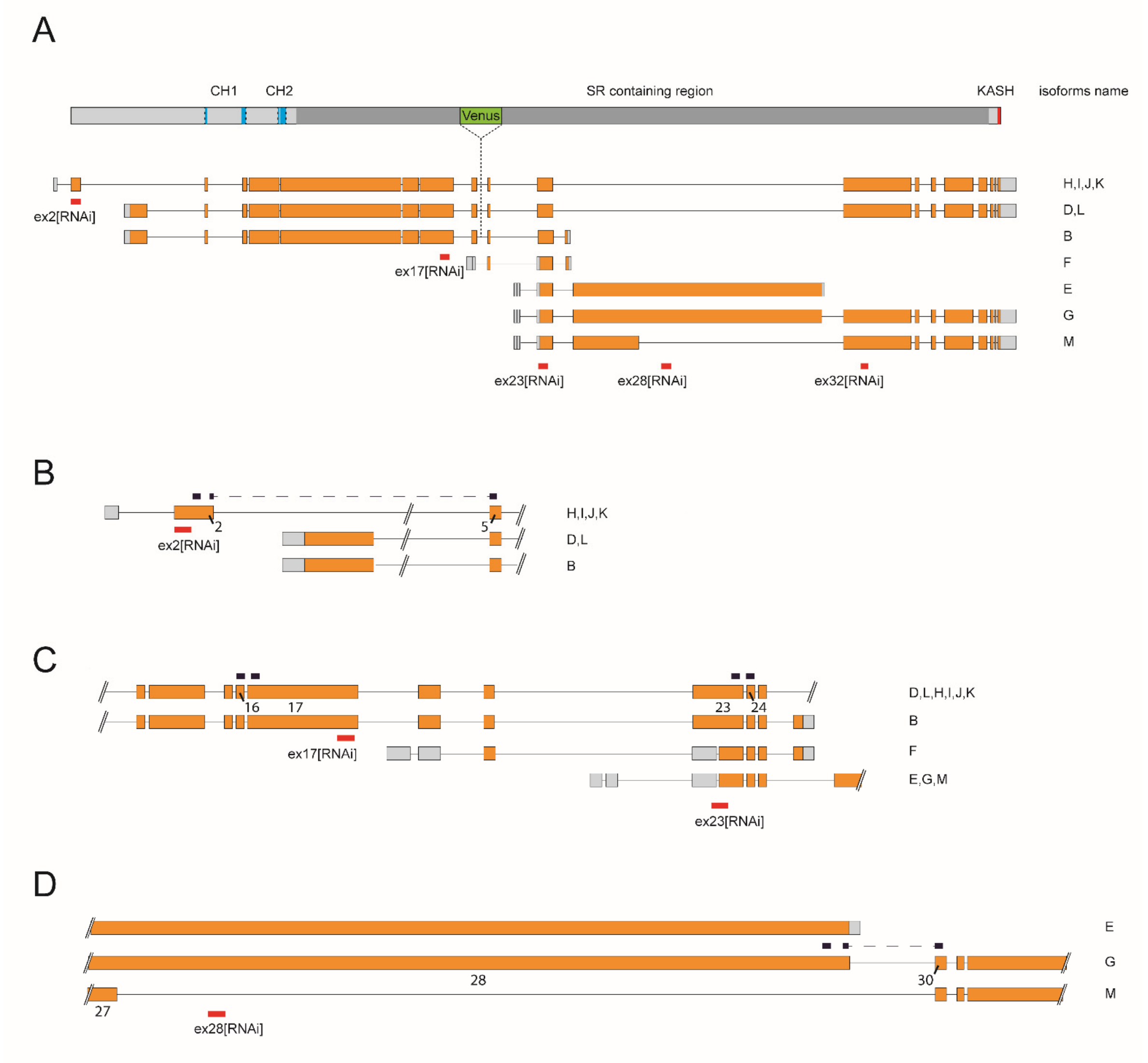

Figure S1: RT-qPCR primers position with respect to RNAi target exons. (**A**) Schematic representation of Msp300 isoforms as in Figure 1. (**B-D**) Close-ups of the exons targeted by RNAi and their immediate neighbours showing the position of the ex[RNAi] in red and of the primer couples used to assess the expression levels of the RNAi targeted exons in black.B-Close-up of the exons 1 to 5 showing the position of ex2[RNAi] (in red) and of the primer couple ex2-5 used to assess the level of ex2 expression by RT-qPCR. (**C**) Close-up of exons 13 to 27 showing the position of ex17[RNAi] and ex23[RNAi] as well as the primer couples ex16-17 and ex23-24. (**D**) Close-up of exons 27 to 32 showing the position of ex28[RNAi] and of the primer couple ex28-30.

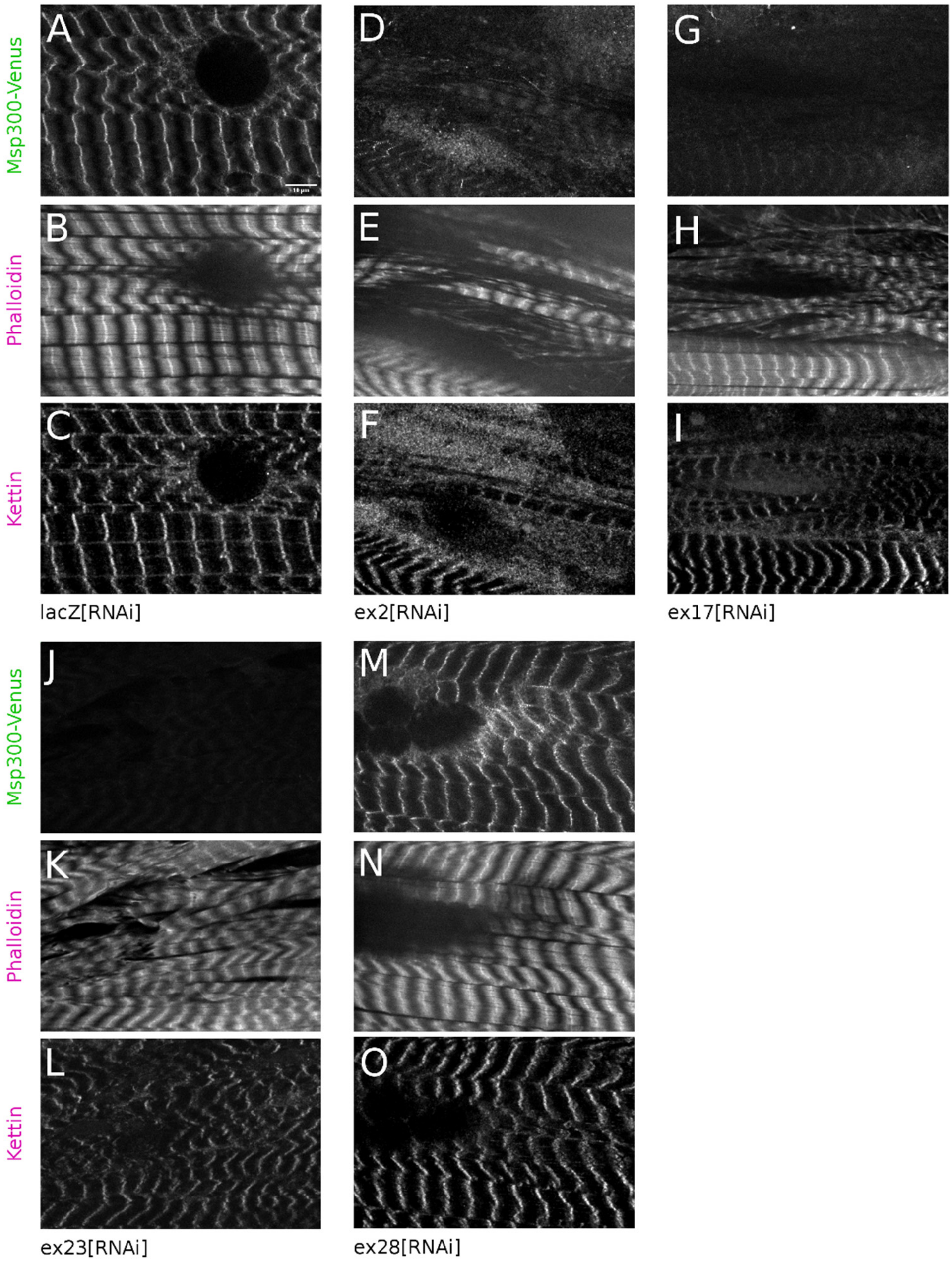

Figure S2. Msp300-Venus, phalloidin and Kettin localisation after isoforms depletion by RNAi. Single channel of overlays from Figure 5 showing the localisation of Msp300-Venus (**A,D,G,J,M**), phalloidin (**B,E,H,K,N**) and Kettin (**C,F,I,L,O**). (**A-C**) lacZ[RNAi], (**D-F**) ex2[RNAi], (**G-I**) ex17[RNAi], (**J-L**) ex23[RNAi] and (**M-O**) ex28[RNAi]. For a full description of all RNAi used, channels corresponding to ex23[RNAi] muscle 4 are shown here. Following ex23[RNAi], Msp300-Venus is completely lost, validating the strength of this RNAi to deplete all CH+ Msp300. Scale bar on lacZ[RNAi] panel A: 10 μm.

Table S1. RT-qPCR primers sequences.

Name	Sequence
ex2-ex5_F	GAGGAGTTCGGTCCTTCGAC
ex2-ex5_R	CACGTTCTTGCTCCTCTTGC
ex16-ex17_F	TCGGTGACAAGCGGTCGCAG
ex16-ex17_R	CTGACCAGCCTGCTTCTTGGCA
ex23-ex24_F	GTGGGCGATCTCAAGGACAA
ex23-ex24_R	TTTTCGTGCCAGGAGCGTAT
ex28-ex30_F	AATACCGGTGCGGTACGAC
ex28-ex30_R	CTTGCGTAATTAGATCCTTCTGG
tbp-1_F	AGTGGTGCTGCCCATGACGC
tbp-1_R	CAGGCACGGGCCAGCAATGT
